# What Shall We Drink?

**Published:** 1875-12

**Authors:** 


### WHAT SHALL WE DRINK?

Whatever it is, it seems evident that it ought not to be impure water. Indeed it is a question whether there is any absolutely pure water to be obtained in a state of nature. It is either contaminated with minerals or vegetables or unclean things. However impregnated, it carries disease and death to those who largely use the poisonous solution.

It is a well-proved fact that the water of malarious regions contains the germs of the agues which infest these localities. The most rigid abstainer from water, suffers the least. Typhoid fever would soon vanish from the world, if only pure water were used for cooking and drinking.

There is probably no drug in the universe as potent for the healing of diseased bodies, as absolutely pure water. So wonderful are its effects that doctors who have employed it declare it to be almost a specific for many diseases, especially for complaints of the stomach.

We are in the habit of speaking with enthusiasm of the delight experienced in delicious draughts of pure water, when the truth is that not one person in one thousand ever tasted pure water. Water is never pure unless rendered so by art. All springs and wells and streams are contaminated and poisoned. The water which decends in rain drops, holds the smoke and dust of the upper air. If you evaporate rain-water to dryness and examine the very large amount of residuum under a microscope, you will find in it hair, and excrement, and insect-germs, and soot, and feathers, and vegetable fibres, and a host of other horrible things. You will find that, though you filter it over and over again this water from the clouds will still be impure, and only less impure than the water of wells, and pools, and streams. Whatever water you examine, you will find it unclean, and you will come to the conclusion that disease can be as surely and as speedily introduced into the system by this means as by bad air or bad food.

What shall we do then? Shall we give up the use of water as a beverage? Shall we conclude, with the old toper, that water is worthless, except for purposes of navigation? The better way will be to see if we cannot devise some means to purify the water used for cooking and drinking. Melted ice is far purer than is water which has never been frozen, because water in freezing deposites much of its impurity. Boiled water is purer than unboiled, because the great heat destroys insect-life; while earthy matters are precipitated in cooling. Still, boiled water is not by any means pure water. Filtered water is not pure water, because much deleterious matter may be held in such complete solution as to pass through the filter. Chemists, who use paper for filtering some liquids, show how impure the most carefully filtered water may be.

There is only one way to purify water, and that is to vaporize it, and then to condense the vapor. This gives as a product, pure water. But this pure water will not remain pure, if left exposed a single hour. It must be sealed securely from dust and air, and all sources of contamination.

The time will come when every house will have its apparatus for the manufacture of pure water. When that day comes, some of our most fearful diseases will be banished from the earth. Here is a field for invention. Here is an opportunity for some genius to provide a boiler with a condenser-attachment, for stoves and ranges, which will keep up the process of distillation whenever the fire is in operation, and thus provide pure water in abundance for the uses of the household. Until this is done, the man who will start a distillery for the purpose of providing pure water for the public, will revolutionize public health and make a fortune.

